# Rapidly Progressive Buschke‐Löwenstein Tumor Managed With Wide Local Excision and Healing by Secondary Intention: A Case Report and Literature Review

**DOI:** 10.1155/cris/4587993

**Published:** 2026-10-20

**Authors:** Sabrina E. Chin, Natalia Rojas Castro, Henry J. Lujan

**Affiliations:** ^1^ Department of Colon and Rectal Surgery Jackson South Medical Center Miami Florida USA; ^2^ Department of Surgery School of Medicine St. George’s University True Blue Grenada sgu.edu; ^3^ School of Medicine and Health Sciences University of Rosario Bogotá Colombia urosario.edu.co

**Keywords:** Buschke-Löwenstein tumor, case report, perianal disease, rapid progression, secondary intention healing

## Abstract

Buschke‐Löwenstein tumor (BLT) is a rare, locally aggressive anogenital tumor associated predominantly with low‐risk human papillomavirus (HPV) types 6 and 11. BLT is classically described as a slow‐growing lesion predominantly affecting middle‐aged men who are immunocompromised; cases of rapid progression are rarely reported. We present the case of a 23‐year‐old male with a progressively enlarging perianal mass over 1 year that was accompanied by rectal pain and intermittent bleeding. He reported prior unprotected heterosexual intercourse, though serologic testing for HIV, syphilis, and hepatitis B/C was negative. CT imaging revealed a fungating lesion entirely distal to the dentate line without evidence of internal extension. Subsequently, the patient underwent wide local excision with electrocautery fulguration, and the wound was managed with healing by secondary intention. Histopathology revealed clear margins around a low‐grade squamous intraepithelial lesion (LSIL) without high‐grade dysplasia or carcinoma; in situ hybridization (ISH) confirmed HPV types 6 and 11, without detection of high‐risk HPV. A review of the literature on BLT epidemiology, histopathology, differential diagnosis, and management is presented. This case illustrates that BLT can progress rapidly in young, immunocompetent individuals and highlights that healing by secondary intention following wide local excision is a viable option in appropriately selected cases.

## 1. Introduction

The Buschke‐Löwenstein tumor (BLT), also known as giant condyloma acuminatum, is a benign exophytic mass that often exhibits aggressive local invasion of the anogenital region. First described in 1896 by Buschke and then further characterized by Buschke and Löwenstein in 1925, this slow‐growing tumor has a cauliflower‐like appearance [1]. It most commonly arises in the anogenital region and is associated with bleeding, pruritus, discharge, and pain [1]. The average patient is 45 years of age, with a 2:1 male predominance [1]. BLT is most strongly associated with low‐risk human papillomavirus (HPV) types 6 and 11, but additional risk factors include chronic irritation, inadequate genital hygiene, immunosuppression, and diabetes [2].

Currently, wide local excision with negative margins is the recommended treatment, with skin flaps or musculocutaneous flaps employed depending on the size of the resulting defect [1]. We report a case of BLT in a young, immunocompetent male who exhibited unusually rapid progression, which was treated with wide local excision alone, and managed with wound healing by secondary intention. This case report has been reported in accordance with the CARE 2013 guidelines [3].

## 2. Case Presentation

A 23‐year‐old male patient presented to the emergency department (ED) with a perianal mass that appeared and increased in size in the past year, with symptoms worsening in the months preceding presentation in the ED. He reported rectal pain and occasional bleeding from the mass. The patient reported unprotected sexual intercourse with a sex worker prior to the appearance of the mass. The patient denied sex with men, anal receptive intercourse, and prior sexually transmitted infections. He reported no family history of colorectal cancer. His HPV vaccination status was unknown.

On initial presentation, the vital signs were within normal limits. The physical exam revealed two large exophytic perianal masses (Figure 1). The remainder of the cardiovascular, pulmonary, and abdominal exams were unremarkable.

**Figure 1:**
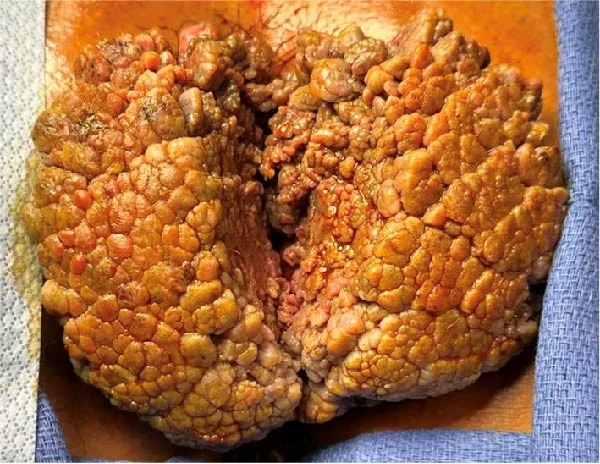
Two large exophytic perianal masses.

Sexually transmitted infections were tested for via serologic testing. HIV antigen/antibody testing was negative. Hepatitis A IgM, hepatitis B core IgM, and hepatitis B surface antigen were negative. Hepatitis C antibody screening was negative. Rapid plasma reagin (RPR) was nonreactive.

CT of the abdomen and pelvis with IV contrast showed a fungating, heterogeneous enhancing mass below the anal sphincter in the anal region at midline (Figure 2). Abnormal tissue in the region of the rectum was absent. No enlarged lymph nodes were seen in the pelvis.

**Figure 2:**
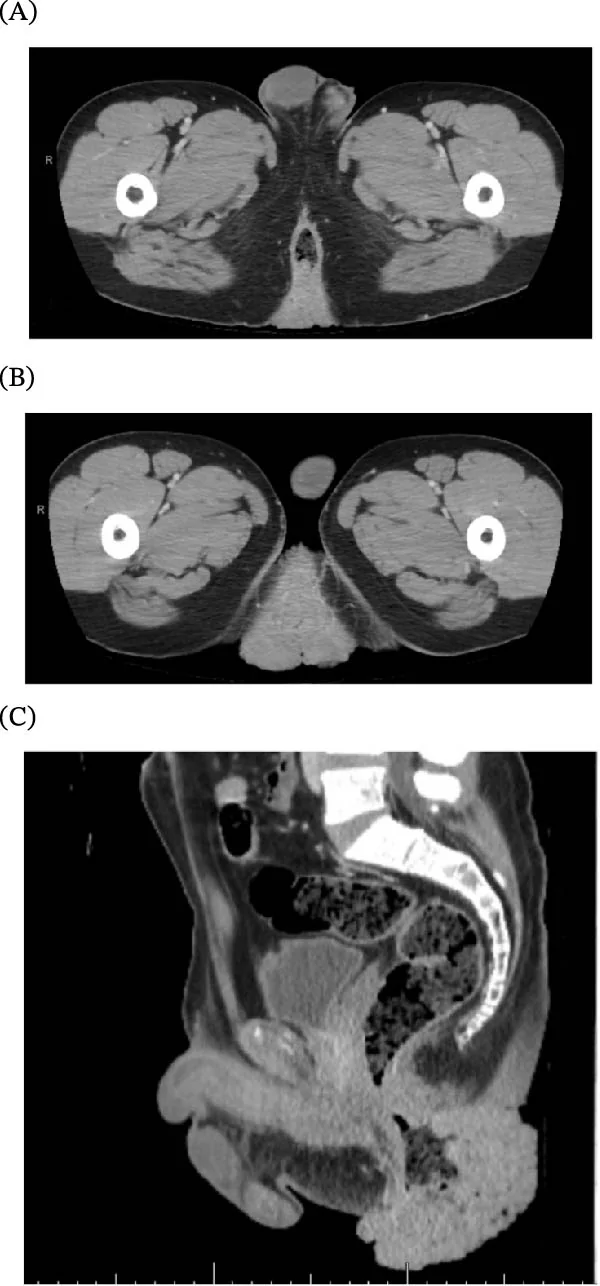
Computed tomography (CT) with contrast of fungating heterogeneous enhancing mass below the anal sphincter in the anal region at midline. (A) Axial view above the anal verge. (B) Axial view below the anal verge. (C) Sagittal view of the mass.

### 2.1. Treatment

Based on CT findings and the location of the mass entirely distal to the dentate line, wide local excision was chosen as the method of removal. In the prone jackknife position, the two perianal masses were excised circumferentially with scissors. All sites and lesions were fulgurated with electrocautery and then thoroughly irrigated. Each mass measured ~8 cm × 10 cm, with minimal satellite lesions. Digital rectal examination and anoscopy revealed no internal lesions. Gross pathology revealed multiple gray‐tan, focally disrupted, cauliflower‐like soft tissues measuring up to 12 cm × 8.5 cm × 4.5 cm in aggregate. Given the absence of sphincter involvement, the decision was made to allow healing by secondary intention.

### 2.2. Diagnosis

Microscopic examination showed a low‐grade squamous intraepithelial lesion (LSIL) with acute inflammation, consistent with a giant condyloma of Buschke‐Löwenstein (Figure 3A,B). The specimen was negative for high‐grade dysplasia or carcinoma, and the margins were free of disease. In situ hybridization (ISH) for HPV types 6 and 11 was strongly positive (Figure 3C). The ISH for high‐risk HPV was negative (Figure 3D). p16 immunohistochemistry was not performed due to the absence of high‐grade dysplasia or carcinoma and negative ISH for high‐risk HPV.

**Figure 3:**
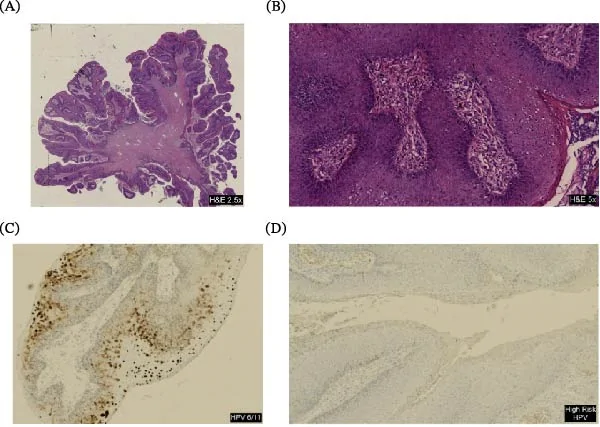
Histopathologic features. (A) Low‐power view demonstrating exophytic, papillomatous architecture, acanthotic squamous epithelium. Absence of stromal invasion, and margins are free of disease. (B) Low‐power view demonstrating low‐grade squamous epithelium with acute inflammation; Koilocytic change is demonstrated in the superficial epithelial layers. (C) In situ hybridization for HPV types 6 and 11 (5x). (D) In situ hybridization for high‐risk HPV.

Based on clinical and imaging findings, BLT was the primary preoperative consideration. Verrucous carcinoma (VC) and squamous cell carcinoma (SCC) were excluded on final pathology by the presence of koilocytic changes, HPV 6/11 positivity on ISH, an intact basement membrane, and clear surgical margins.

No diagnostic challenges related to access, financial constraints, or cultural factors were encountered.

### 2.3. Outcome

The patient was followed up on postoperative days (POD) 1, 4, 18, 32, and 46 for wound assessment and surveillance for recurrence, with photographic documentation.

POD 1 (Figure 4A): Normal healing. Gauze was reapplied.

**Figure 4:**
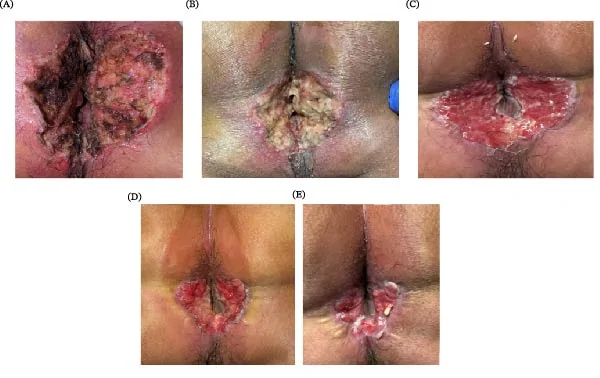
Postoperative wound assessment. (A) Postoperative day (POD) 1. (B) POD 4. (C) POD 18. (D) POD 32. (E) POD 46.

POD 4 (Figure 4B): Normal healing wounds with normal postoperative swelling and no signs of infection. Wet‐to‐dry dressingchanges for wound care was recommended.

POD 18 (Figure 4C): Granulating wounds were seen with no signs of infection. Wet‐to‐dry dressing changes for wound care was recommended.

POD 32 (Figure 4D): The anal derm was intact circumferentially. Two large wounds with hypertrophic granulation tissue. Mild perianal dermatitis was present. There were no signs of erythema or infection. Silver nitrate was used to reduce the granulation tissue burden, and a repeat treatment in 1 week was recommended. Topical zinc oxide or menthol/zinc oxide ointment was recommended at bedtime for dermatitis.

POD 46 (Figure 4E): Inspection showed a significant reduction in the size of the wounds bilaterally, although hypertrophic granulation tissue persisted. Silver nitrate was applied to the right side, and a repeat treatment was recommended in 2 weeks for the hypertrophic granulation tissue.

The patient did not return for further follow‐up after POD 46.

## 3. Discussion

This case challenges the conventional characterization of BLT as indolent, demonstrating that BLT can progress rapidly even in a young, immunocompetent patient without traditional risk factors. BLT, alternatively known as giant condyloma acuminatum, is a locally destructive tumor of the anogenital region without lymphatic, vascular, or neuronal invasion [4]. It is frequently reported in males, typically in the 4th to 6th decades [2]. The quadrivalent and 9‐valent HPV vaccines both cover HPV types 6 and 11 and have significantly reduced the prevalence of anogenital warts in vaccinated populations [5].

Nevertheless, BLT has been well‐documented in individuals with sexual behaviors associated with HPV exposure, including early sexual activity and multiple partners. Host‐related factors—immunosuppression, smoking, socioeconomic determinants, and substance abuse—may further predispose to disease development [2]. Although “giant” is central to the diagnosis, it lacks a standardized definition in the literature. To address this, Davis et al. [6] proposed defining BLT as an anogenital verrucous mass larger than 5 cm in total diameter and occupying greater than one‐third of the circumference of an anogenital organ. Additionally, they offered a grading system based on gross size prior to excision to improve the understanding of BLT and guide treatment planning [6].

A prolonged persistence of BLT over years to decades is commonly reported—the longest documented case being a penile lesion spanning 44 years. However, rapid expansion over weeks has been reported during pregnancy and in a postmenopausal woman [6]. Our patient’s clinical course also defies the typical growth pattern, with the lesion onset within months of engaging in high‐risk sexual behavior and marked progression over the 3 months preceding presentation. Clinically, patients may present with a range of symptoms related to progressive local disease, such as pain and discomfort, bleeding, mass lesions, and discharge or draining abscesses in the anal region [7].

BLT is histologically characterized by koilocytosis, parakeratosis, hyperkeratosis, and acanthosis [8]. Zhang et al. [7] identified intraepithelial neutrophilic inflammation, dyskeratosis, and keratin cysts as potential features that could distinguish BLT from a true condyloma. Histopathological examination showed uninvolved surgical margins and no evidence of high‐grade dysplasia or carcinoma, confirming the diagnosis of BLT in our patient. The primary causative agent of BLT is low‐risk HPV (6 or 11) and rarely high‐risk HPV strains such as 16 and 18 [4]. ISH for our patient was strongly positive for HPV types 6 and 11 and negative for high‐risk HPV types, providing molecular confirmation of low‐risk HPV etiology. p16 immunohistochemistry was not pursued given the absence of high‐risk HPV and high‐grade dysplasia. Zhang et al. [7] reported invasive SCC in 50% of their multi‐institutional BLT cases. Among the subset that underwent HPV genotyping, 10 of 13 SCC‐harboring cases were associated with high‐risk HPV and/or p16 positivity [7]. In our case, low‐risk HPV infection, a relatively short disease duration, and the absence of malignancy suggest more favorable biologic behavior.

Beyond confirming the diagnosis, histopathologic and molecular findings are essential for distinguishing BLT from morphologically similar entities. BLT and VC are still used interchangeably, even though they are distinct entities. VCs are exophytic tumors that are rarely associated with HPV infections, characterized histologically by thick, club‐shaped islands of well‐differentiated, cytologically uniform squamous epithelium with enlarged spinous cells and the lack of koilocytosis. Additionally, the invasion pattern of VC—described as well‐defined, bulbous, pushing borders that invade the stroma—is not typically observed in BLT [7, 9, 10]. In our case, strong HPV 6/11 positivity on ISH and the presence of koilocytic changes are inconsistent with VC.

Consistent with our patient’s histopathological findings, BLT is distinguished from SCC by an intact basement membrane and the absence of metastatic potential [2].

Currently, there is no clear consensus regarding the optimal management of BLT as its size, recurrence, and degree of invasion present significant therapeutic challenges. However, wide local excision remains the most commonly reported treatment and has been associated with favorable outcomes [11]. In cases of large defects, skin flaps or grafts, including fascio‐cutaneous V‐Y advancement flaps and bilateral keystone perforator skin flaps, may be used for reconstruction [10, 12]. V‐Y advancement flaps are suitable for large defects in the gluteal region as the tissue is well‐vascularized and the V‐Y advancement design permits primary donor‐site closure [13]. Our patient’s lesion was entirely distal to the dentate line, and as such, the surgical defect was managed with healing by secondary intention, with satisfactory wound progression observed at 6.5 weeks. However, the patient was subsequently lost to follow‐up before complete healing could be documented, and the development of complications such as tumor recurrence, anal strictures, mucosal ectropion, or fecal incontinence could not be assessed. Compared with skin grafts or flaps, healing by secondary intention avoids donor site morbidity and flap failure, albeit at the cost of a longer healing timeline. Colostomy creation in anticipation of abdominoperineal resection or to facilitate healing by reducing surgical site contamination has been reported in cases of extensive local disease with sphincter or rectal involvement [11, 14]. In some of these cases, subsequent histopathology revealed transformation of BLT to SCC, prompting adjuvant chemotherapy (fluorouracil, cisplatin) and/or radiation [11, 15]. Alternative therapies, such as topical photodynamic therapy, imiquimod cream, and CO2 laser ablation, are less widely utilized [6, 16–19].

BLT is often described in the literature as having a high recurrence and mortality rate. A 42‐case series by Chu et al. [20] reported an overall recurrence rate of 66% (28/42) and an overall mortality of 21% (9/42). Among patients treated with radical surgery, those who developed recurrence had a longer average duration (9.6 years vs. 2.8 years), suggesting an association between the prolonged disease course and recurrence [20]. In contrast, a multi‐institutional series of 38 cases by Zhang et al. [7] reported a lower recurrence rate of 23.7% (9/38) and an overall mortality rate of 10.5% (4/38). Although Zhang et al. [7] report lower rates of recurrence and overall mortality across a wide follow‐up range (1–207 months), the relatively short median follow‐up (23 months) may capture only early posttreatment outcomes. Conversely, Chu et al.’s [20] findings likely reflect disease behavior over prolonged clinical courses. Both case series are limited by their retrospective design and heterogeneous follow‐up periods. In our review of both series, reported mortalities were attributable to comorbidities (e.g., coronary artery disease), treatment complications (e.g., peritonitis secondary to colostomy failure), or malignant transformation (e.g., carcinoma with nodal metastasis) rather than BLT directly [7, 20]. This observation is consistent with Davis et al.’s [6] comprehensive review, which found a similar pattern across multiple additional case series. In this context, the clinical and pathologic profile of our patient—defined by low‐risk HPV, a short disease course, clear margins, and lack of malignant degeneration—aligns with cases reported in the literature that have not recurred or required escalation of treatment.

The primary limitation of this case is that the patient was lost to follow‐up after POD 46. As a result, complete wound healing or the absence of recurrence could not be confirmed. Given that BLT has a reported recurrence rate of 23.7%–66%, the possibility of local recurrence and possible malignant development cannot be excluded. Therefore, the favorable early wound trajectory of healing by secondary intention should be interpreted with caution as the results preclude a definitive assessment of long‐term outcomes. Additionally, as a single case report, the generalizability of the surgical approach and results is limited. Comments on the failure or lack of HPV vaccination are limited due to the unknown vaccination status of the patient. Finally, although high‐grade dysplasia or carcinoma and negative ISH for high‐risk HPV clinically justified not performing p16 immunohistochemistry, this staining would have provided additional information.

This case supports several practical points for the management of perianal BLT. The distal location of the lesion and the absence of sphincter involvement permitted a conservative nonreconstructive approach with healing by secondary intention, avoiding donor site morbidity and flap failure associated with flap closure. Satisfactory wound progression was observed through POD 46, suggesting that tissue‐sparing management may be a viable option in select cases of BLT. However, confirmation of complete wound healing, exclusion of late recurrence, and assessment of complications of the excision were not possible because the patient was lost to follow‐up after POD 46. The integration of ISH and histopathological evaluation proved essential for confirming low‐risk HPV status, enabling differentiation from VC and exclusion of SCC. These findings had a direct impact on management as the presence of high‐risk HPV or malignant transformation would have warranted adjuvant chemoradiation. Finally, the rapid progression of this lesion in a young, immunocompetent male suggests that tissue diagnosis—rather than aggressive growth alone—should be used to infer malignant potential.

The present case adds to the paucity of literature documenting the rapid progression of BLT in young, immunocompetent individuals. It underscores that BLT should remain in the differential diagnosis for rapidly enlarging anogenital lesions, regardless of the patient’s age or immune status. Additionally, a critical gap in the current evidence base is exposed regarding the BLT heterogeneity of the presentation, growth kinetics, and malignant potential. A multi‐institutional registry with uniform reporting criteria, a standardized size‐based grading system—such as that proposed by Davis et al. [6]—and consensus surveillance protocols are necessary to move beyond case reports towards evidence‐based management guidelines.

## Funding

No funding was received for this research.

## Disclosure

All authors have read and approved this manuscript for submission. All content was reviewed, verified, and approved by the authors, who take full responsibility for the content and integrity of the work.

## Ethics Statement

This case report was prepared in accordance with the ethical principles of the Declaration of Helsinki. As a retrospective report of a single patient’s clinical care, this work does not constitute human subjects research under U.S. federal regulations (45 CFR 46). Consistent with institutional practice, formal IRB review was not pursued for this single‐case report. Clinical data and images were collected as part of routine care and were subsequently used for educational and scientific reporting purposes. Multiple attempts were made to contact the patient to obtain written informed consent for publication; however, the patient could not be reached after being lost to follow‐up. In the absence of obtainable consent, all directly identifiable information has been removed from the text, clinical photographs, and radiologic images in accordance with ICMJE recommendations and HIPAA deidentification standards (Safe Harbor method). The authors affirm that the risk to patient privacy is minimal given the comprehensive deidentification measures employed.

## Consent

See Ethical Considerations.

## Conflicts of Interest

The authors declare no conflicts of interest.

## Data Availability

Data that support the findings of this study are available upon request from the corresponding author. The data are not publicly available due to privacy or ethical restrictions.
